# Maxillary protraction with rapid maxillary expansion and facemask versus skeletal anchorage with mini-implants in class III patients: a non-randomized clinical trial

**DOI:** 10.1186/s40510-019-0288-7

**Published:** 2019-09-02

**Authors:** Ricardo Alves de Souza, José Rino Neto, João Batista de Paiva

**Affiliations:** 1 School of Dentistry, University of São Paulo, São Paulo, Brazil; 2 Southwest Bahia State University, Jequié, Bahia Brazil; 3 Department of Orthodontics, School of Dentistry, University of São Paulo, Av. Prof. Lineu Prestes, 2227, Butantã, São Paulo, SP 05508900 Brazil

**Keywords:** Angle class III malocclusion, Maxillary retrusion, Orthodontic anchorage procedures, Facemask

## Abstract

**Background:**

The use of skeletal anchorage devices for maxillary protraction in patients with class III malocclusion due to deficiency in the middle third of the face has been shown to be a promising approach to treatment of these patients. The aim of this study was to evaluate the treatment of class III patients with maxillary retrusion, using orthodontic mini-implants (MI) associated with intermaxillary elastics in comparison with the rapid maxillary expansion and facemask protocol (RME/FM).

**Methods:**

In this prospective non-randomized clinical trial, the sample of 24 participants between 7 and 12 years of age (median age of 10.0 years and interquartile range = 3.0 years), at the stage prior to the pre-pubertal growth spurt, was divided in two groups. In group facemask (FM) (*n* = 12), the individuals received orthopedic treatment with RME/FM. In group MI (*n* = 12), two mini-implants were inserted in the region close to the maxillary first molar roots, and the other two in the region of the mandibular canines. Initial and final lateral teleradiographs were taken for cephalometric evaluation of all the cases. Statistical analysis included the Mann-Whitney, Wilcoxon, and Fisher’s exact tests. The level of significance was 5% (α = 0.05).

**Results:**

Improvement was verified in the facial profile and occlusion of the participants, showing advancement of the maxilla in the two groups, with significant differences (*P* ≤ 0.05) between T0 and T1 in the following measurements: SNA, ANB, Wits, Co-A, Co-Gn, NAP, A-Npog, overjet, and molar relationship. There was no statistically significant intergroup difference (*P* > 0.05) in the cephalometric measurements evaluated, but the time of treatment was significant, and was faster for group MI.

**Conclusions:**

The protocol with mini-implants may be an option for the correction of Class III due to maxillary deficiency.

## Background

Over the course of several decades, patients with class III malocclusion due to deficiency of the middle third of the face, at the stage of growth, have been treated by means of maxillary protraction with a facemask presenting satisfactory results [1–12], when the patient cooperates with the use of the extra oral appliance [13, 14]. During the last few years, with the justification that it depends less on the patient, and that it eliminates the undesirable results, for example, mandibular rotation and dental effects, some authors have replaced maxillary expansion and the facemask with orthodontic miniplates for skeletal anchorage associated with intermaxillary elastics, and have obtained significant results in maxillary protraction for treatment of class III patients [14–18]. However, the great disadvantage of this protocol is the need to perform surgical interventions in children to insert and remove the miniplates, leading to family members being reluctant to accept this option, and making it difficult to consolidate this technique as a routine treatment in orthodontic clinics.

Reports have shown a 92.5% success rate for miniplates placed by oral surgeons, and over 80% of the placement surgeries were considered very easy to moderately simple. Nevertheless, approximately 20% of the cases analyzed presented some type of problem, such as for example size of the device incompatible with the patient’s anatomy or excessive bone growth over the miniplates during removal, in addition to the surgical time spent in cases of low bone density. The occurrence of moderate to severe swelling was also cited in approximately 45% to 25% of cases, respectively, and even a case in which the screws were partially left behind within the bone. The authors confirmed that general sedation was the preferred option in cases in which four miniplates were scheduled for placement in children for the purpose of orthopedic traction [19].

The possibility of using mini-implants as anchorage for maxillary protraction was demonstrated, by inserting these devices in the palate, in association with a modified Hyrax type expander. The authors considered the side effects minimal after maxillary protraction in class III patients, when compared with patients with dental anchorage. There was less opening of the mandibular plane and less descendant movement of point A. However, both groups were treated with the use of a facemask, and depended on a higher level of patient cooperation with the use of the extraoral appliance [20].

Therefore, the aim of this study was to test whether conventional orthodontic mini-implants inserted in the maxilla and mandible, associated with intermaxillary elastics would serve as anchorage for protracting the maxilla in class III patients with deficiency of the middle third of the face, and compare the results with those of patients treated with rapid maxillary expansion and facemask.

## Methods

### Participants

The total sample used in this study consisted of 24 individuals with a median age of 10.0 years (interquartile range = 3.0 years), of whom 11 were of the male (45.8%), and 13 of the female sex (54.2%). The patients were divided into two groups: facemask (FM) with 12 individuals, median age 8.0 years (interquartile range = 4.0 years), of whom six (50%) were of the male and six (50%) of the female sex, who received conventional treatment with rapid maxillary expansion and facemask. The mini-implant group (MI) with 12 individuals, median age 10.0 years (interquartile range = 1.8 years), of whom four (33%) were of the male and eight (67%) of the female sex, treated with the use of mini-implants as anchorage for maxillary protraction and intermaxillary elastics.

The inclusion criteria were that all patients had to present angle class III malocclusion due to deficiency of the middle third of the face, and that the pre-pubertal growth spurt had not yet occurred; this was evaluated by means of carpal radiography. The diagnoses of patients were confirmed by means of clinical parameters, manipulating the mandible of children in centric relationship to avoid deviation of the mandible in the anterior direction, and also in centric occlusion, to verify the molar relationship and presence of negative overjet incisor end-to-end relationship. Diagnosis was also made by facial analysis and cephalometric parameters of the individuals, finding the presence of straight or concave profile, Wits appraisal smaller than or equal to 2 mm and ANB smaller than 1°. The patients could not present systemic problems, or any type of syndrome, pseudo-class III or muscle dysfunctions, and must not have undergone any previous orthodontic treatment. Presence of interradicular spaces for insertion of mini-implants was an inclusion criteria for the group MI.

### Treatment protocol

The treatment of participants of group FM was performed by means of rapid maxillary expansion with a modified Hyrax expander, presenting orthodontic bands on the permanent molars. In addition, 0.9 mm orthodontic wire was welded on the bands on the vestibular and palatine surfaces, with extension up to the mesial region of the primary canines, where a hook was placed for adapting the elastics. The expander was activated daily with one-fourth of a turn in the morning and the other at night, until the palatine cusps of the maxillary molars were close to the top of the vestibular cusps of the mandibular molars. This situation occurred in a period of approximately 8 to 12 days of activation. Right after this, the expander screw was locked and a Petit facemask—with heavy ½′′ (inches) extraoral elastics, with force of approximately 400 grams (g)—was fitted for maxillary protraction for a period of 14 to 16 h per day. The patient and family members were instructed about the regular use of the extraoral appliance and that the elastics had to be changed every day. At each monthly consultation, the patient was constantly motivated to use the facemask and elastics, and at all times to follow the initial instructions. The extraoral appliance was used until correction of the negative overjet and improvement in facial profile (2 mm overcorrection). After this period, the orthodontic treatment was initiated with fixed appliances to conclude the case. All the patients of this group were treated orthopedically by only one orthodontist, and no desistance occurred after treatment began.

**Fig. 1:**
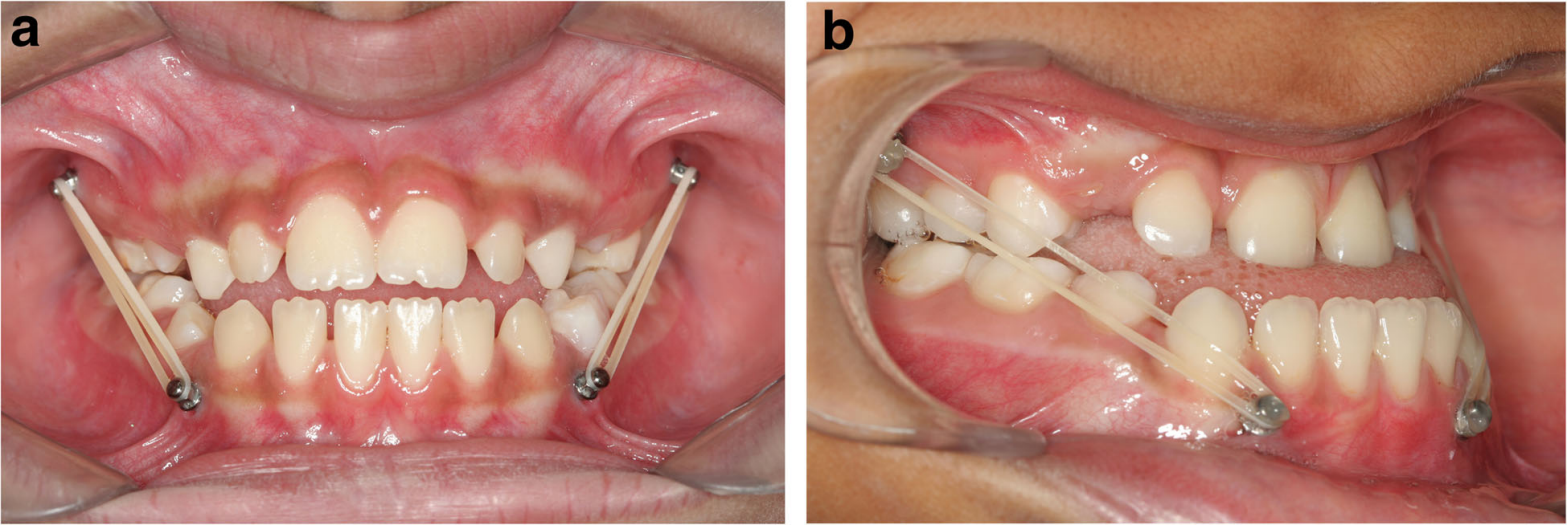
Patients of group MI with mini-implants and intermaxillary elastics inserted. Verify the difference in the position of the mini-implants in **a** and **b** resulting from the need for inter-radicular space

In group MI, the patients received four conventional orthodontic mini-implants, 10 or 8 mm long, with a transmucosal section of 2 mm, and 1.5 mm thick, inserted in the mesial regions of teeth 16 and 26. Another two were inserted in the distal region of teeth 33 and 43, or mesial regions of these same teeth, due to the need for adequate space between the roots, which occurred in four cases. These devices served to enable adaption of the intermaxillary elastics, and make it possible to attempt to produce maxillary protraction (Fig. 1). Topical anesthesia was used for insertion of the mini-implants, and after 1 min, submucosal anesthesia was applied with a short gingival needle in the region that would receive the mini-implants. The mini-implants were preferably inserted in the attached gingiva region, or at the limit with the oral mucosa; however, eight devices had to be inserted in the oral mucosa zone to distance them from the germs and roots of the teeth present in the region of choice. Light ¼′′ intermaxillary elastics were used right after insertion of the four mini-implants for a period of 1 month. These produced an initial force close to 100 g on each side, considering the patient with the mouth in a closed position. As from the second month, medium ¼′′ elastics were used, which resulted in a force of approximately 200 g (with a closed mouth). During the first consultation after insertion of the devices, analgesic was prescribed for the children, and both the children and family members were instructed that the elastics had to be used 24 h a day, removing them only when eating and performing oral hygiene and mini-implant cleaning. At each monthly consultation, the parents were asked whether the children were using the elastics, and motivation for using these accessories was reinforced. Moreover, the orthodontist checked whether oral hygiene and cleanliness of the mini-implants were satisfactory. All the participants of this group were also treated by only one orthodontist until the problem was overcorrected (2 mm). No interruption occurred after the treatment began. The number of lost mini-implants and those that remained throughout all the treatment were analyzed to evaluate the stability of the mini-implants.

**Fig. 2:**
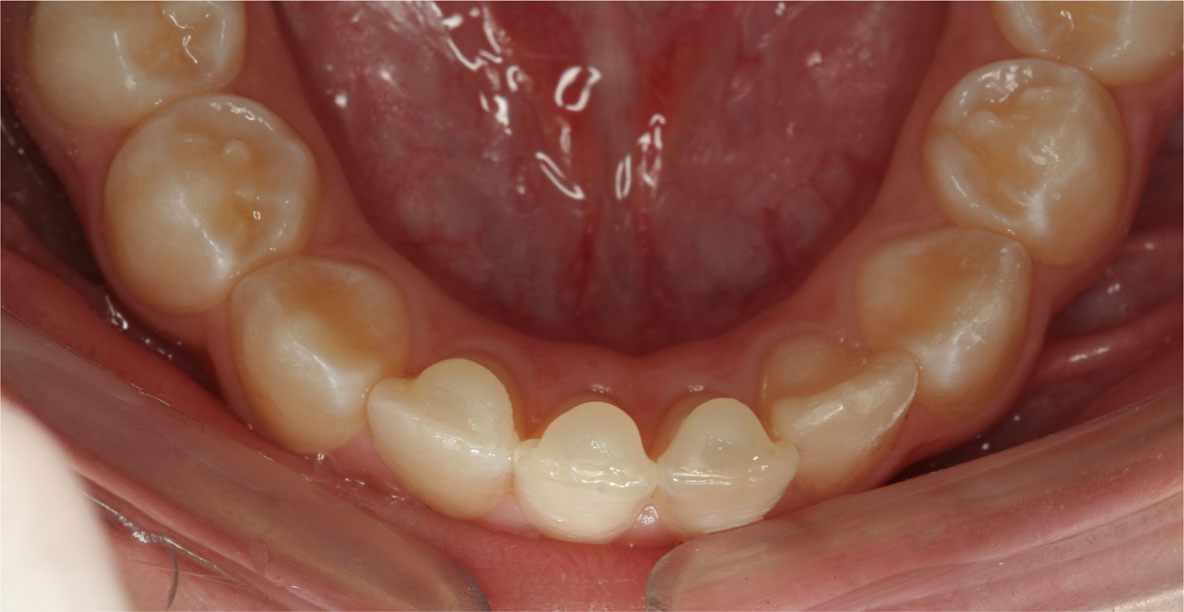
To elevate the bite, stops made of flow resin were inserted on the mandibular incisors

In both groups, when there was locking of the bite in the region of the incisors, a stop made of flow resin was used on the lingual surfaces of the mandibular incisors, to eliminate occlusal interference (Fig. 2).

All the patients were treated at a stage prior to the pre-pubertal growth spurt, and initial (T0) and final (T1) teleradiographs were taken for cephalometric evaluation of the cases. The same cephalometer was used for all the radiographs taken to guarantee standardization of the exams. All the images were taken with the lips relaxed, and in centric occlusion. To prevent bias of data assessment, the evaluator was blinded to the groups to which the teleradiographs belonged. The evaluator was duly calibrated and manually produced all the initial and final cephalometric tracings without knowing to which group the exam pertained. Therefore, the orthodontist who treated the participants was a different professional from the one that evaluated the teleradiographs. To evaluate the intraexaminer error, 20% (percentage) of the teleradiographs were randomly selected and measured again, after a minimum interval of 4 weeks. For evaluating the systematic error, the paired *t* test was applied, and the magnitude of casual errors was calculated by the Dahlberg formula. After analysis, good agreement between the measurements was verified. The errors above 1 mm for linear measurements and above 1.5° for angular measurements were considered significant.

### Cephalometric analysis

For skeletal evaluation, the cephalometric variables used in the study were as follows: SNA (sagittal relationship of the maxilla with the base of the skull), SNB (sagittal relationship of the mandible with the base of the skull), ANB (sagittal discrepancy between the maxilla and mandible), Wits (sagittal relationship between the maxilla and mandible), Co-A (effective length of the maxilla), Co-Gn (effective length of the mandible), and SN.GoGn (facial growth pattern). In the dental evaluation, the following were used: 1.NA (maxillary incisor inclination), 1-NA (protrusion or retrusion of the maxillary incisor), 1.NB (mandibular incisor inclination), and 1-NB (protrusion or retrusion of the mandibular incisor). For facial evaluation, the following were analyzed: NAP, A-NPog, and Sn-Line H (described the degree of convexity and harmony of the facial profile). The overjet, overbite, and molar relation of the participants were also analyzed.

### Statistical analysis

For descriptive analysis of the data, the median and interquartile range were used. The frequency of a specific gender between the groups was tested using the chi-square test or Fisher’s exact test (for cases where the expected frequency was less than five). Due to the small size of the sample, we chose to use only non-parametric inferential analysis. Comparisons between two independent samples were performed by the Mann-Whitney *U* test, and between two paired samples, by means of the Wilcoxon test. The level of significance adopted for all the analyses was 5% (α = 0.05). The data were tabulated and analyzed in the software program IBM SPSS Statistics for Windows (IBM SPSS. 21.0, 2012, Armonk, NY: IBM Corp.).

The pilot study created parameters to determine the number of participants who will be required to compose the study sample, and to verify whether the methodology would be feasible. Four participants with class III malocclusion due to deficiency of the middle third of the face were treated at a stage prior the prepubertal growth spurt. This was performed following the protocol with RME and FM, for comparison with four other children, treated in accordance with the experimental methodology with mini-implants and intermaxillary elastics. After cephalometric evaluation of the cases and statistical analysis by means of the Student’s *t* test, Mann-Whitney test for comparison of independent samples, Wilcoxon test for paired samples, and level of significance of 5%, the preliminary results showed an improvement in the final cephalometric measurements in both the facemask and mini-implant groups. In the intragroup comparison, no statistically significant differences were shown, with the exception of variable SNA that improved significantly in the group treated with mini-implants, in spite of the reduced sample. Sample calculations were made to establish the minimum sample required for each cephalometric variable used in this study, with a view to applying tests for paired samples. To calculate the sample size, we used parameters estimated from a pilot study: pre and post treatment difference in each cephalometric parameter, standard deviations of the differences, test power of 80% (β = 0.20), and error of 5% (α = 0.05). The results pointed out that each group in this type of study should have a minimum of ten patients. This parameter was increased by 15% due to the use of the Mann-Whitney *U* test and to allow for any desistance during the treatments, which generated a sample of 24 participants (12 in group FM and 12 in group MI). The sample calculations were made in the BioEstat Program (version 5.3, Brazil). It was not possible to carry out ideal randomization of the sample, by virtue of the fact that the participants of group MI would necessarily have to present inter-radicular spaces for insertion of mini-implants; therefore, during selection of the cases, all the radiographs were analyzed for possible regions where the mini-implants of all the participants could be inserted. Those who presented space were randomly allocated to group MI until a minimum number of the sample calculation was reached, and all the other individuals were allotted to group FM; since some presented space for mini-implants and some did not, and these in general were younger children and of the male sex. This distribution of patients did not affect the homogeneity of the sample of this research from the aspects of age and sex.

The model of this prospective non-randomized clinical trial was approved by the Ethics Committee; Protocol Number 1.070.768, without change in the judgment after the study began.

## Results

Analysis of the cephalometric measurements in groups FM and MI in T0 showed no significant differences between the groups, indicating that the two groups presented similar clinical characteristics at baseline of the study (Table 1). There was no significant difference between groups FM and MI as regards distribution between the sexes (*P* = 0.529, chi-square test) and median age (*P* = 0.079, Mann-Whitney test). This distribution of patients did not affect the homogeneity of the sample of this research from the aspects of age and sex.

**Table 1:** Intergroup comparisons of initial facial characteristics (T0) of the sample

Variable	Facemask		Mini-implant		*P* value*
	Median	Interquartile range	Median	Interquartile range	
SNA (°)	80.00	4.00	84.00	5.40	0.051
SNB (°)	82.00	2.00	83.00	1.80	0.061
ANB (°)	− 1.00	2.00	− 1.00	4.00	0.458
Wits (mm)	− 7.00	4.50	− 6.50	3.80	0.512
SN-GoGn (°)	32.50	5.80	30.25	5.30	0.102
Co-A (mm)	81.75	3,80	85.75	10.90	0.060
Co-Gn (mm)	112.00	7.50	117.00	11.80	0.367
NAP (°)	− 3.00	2.50	− 2.00	8.40	0.511
A-Npog (mm)	− 1.00	1.50	− 0.50	3.40	0.269
Sn-line H (mm)	5.00	2.30	7.25	3.80	0.087
1.NA (°)	29.00	7.30	29.50	7.90	0.663
1-NA (mm)	6.00	3.80	6.50	5.40	0.640
1.NB (°)	25.00	10.00	28.25	5.80	0.180
1-NB (mm)	5.00	4.30	6.25	2.80	0.190
Overjet (mm)	− 1.00	4.00	− 3.00	4.00	0.350
Overbite	1.00	2.80	0,25	1.90	0.719
MR (mm)	− 2.00	4.00	− 2.00	4.80	0.890

*Mann-Whitney test. The results were expressed as median and interquartile range

**Fig. 3:**
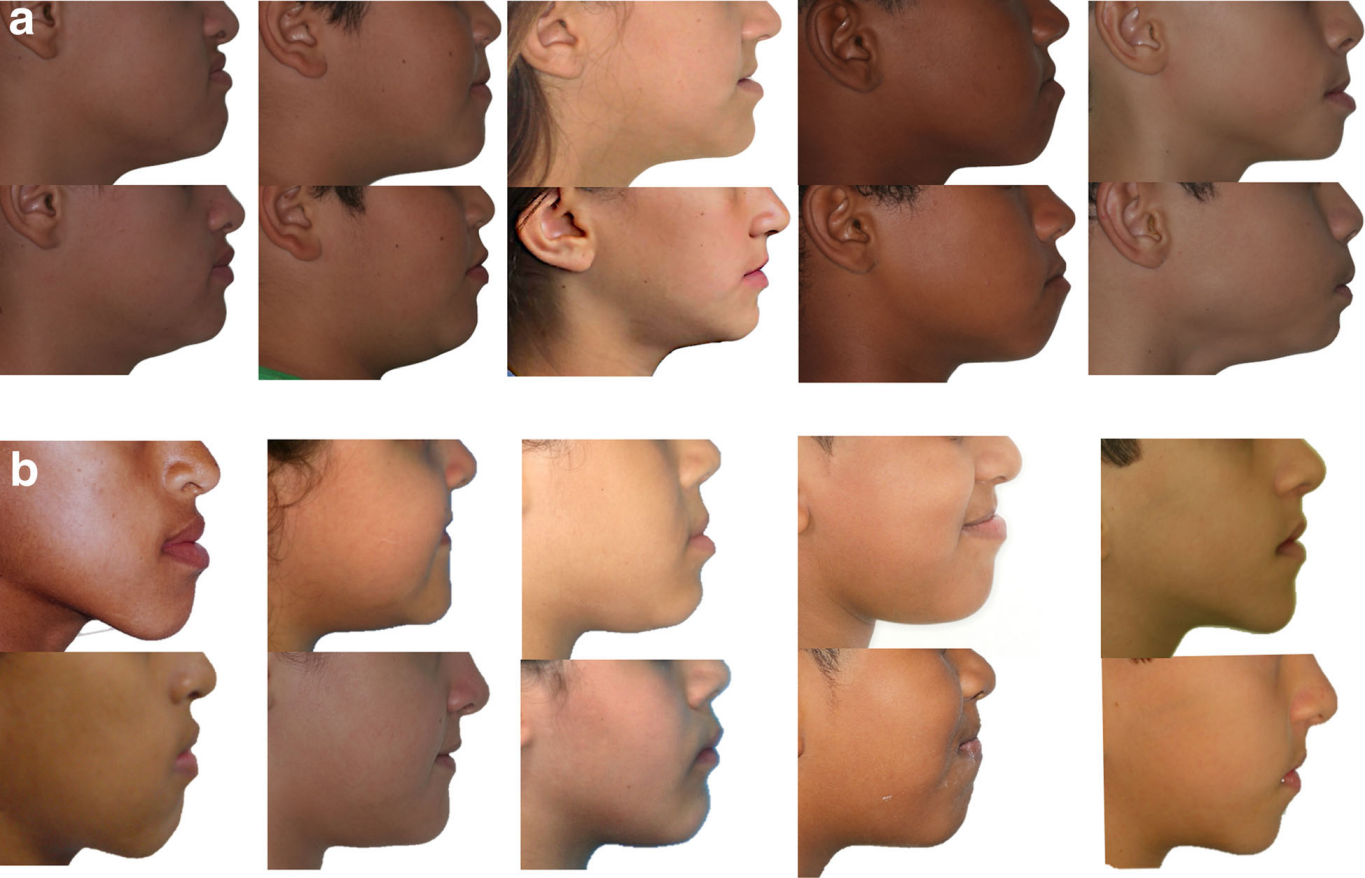
Aspect of the facial profile before and after treatment, of five patients of group MI (**a**) and five patients of group FM (**b**)

**Fig. 4:**
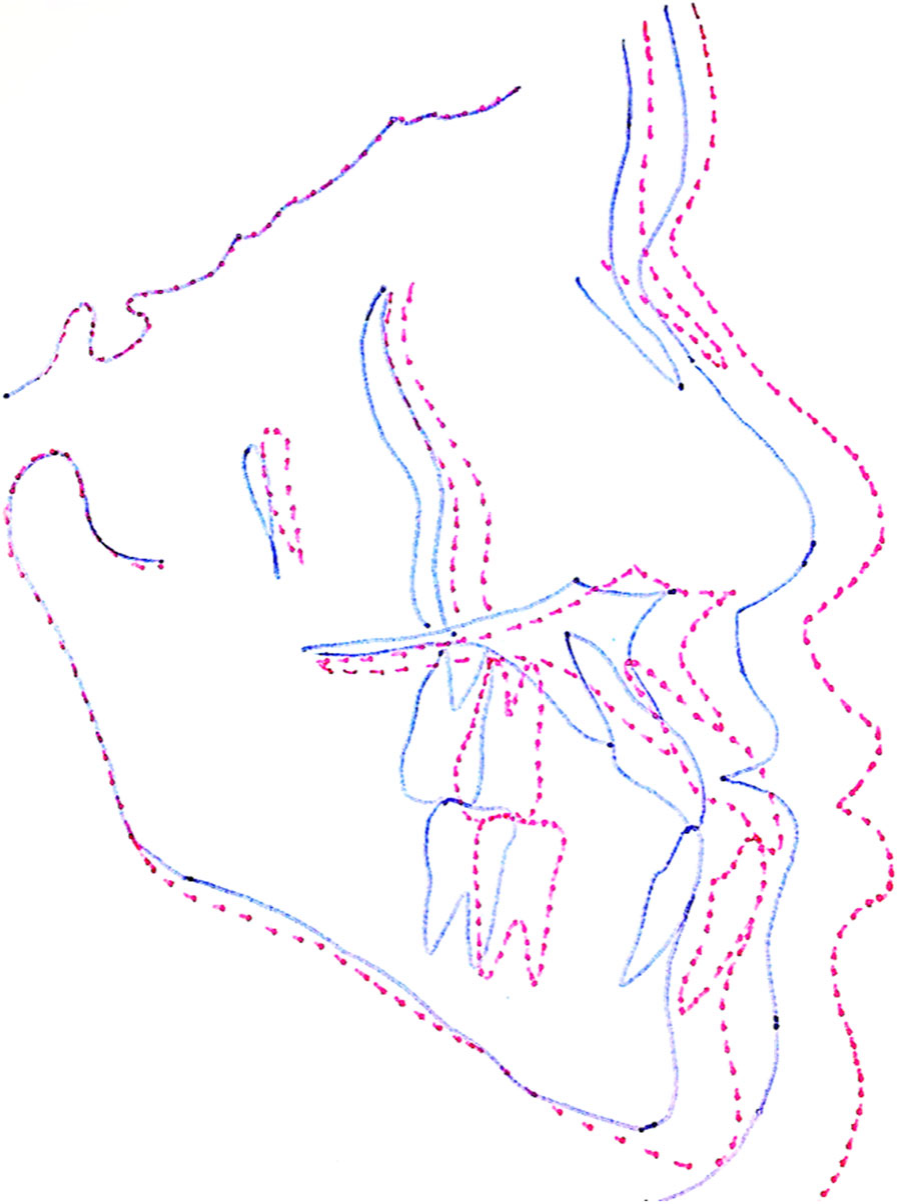
Cephalometric superimposition of a patient of group MI

After treatment with the use of skeletal anchorage with mini-implants, the participants of group MI presented improvement in the facial profile and occlusion, with correction of anterior cross bite, in a manner very similar to that of the patients in group FM. In Fig. 3, it was possible to verify the profile of five participants of group MI and five of group FM, in whom, in general, the maxilla was more protracted than the mandible and there was an increase facial convexity. In Fig. 4, the cephalometric superimposition of one patient of group MI showed a discrete anti-clockwise rotation occurred in the palatine plane, and slight rotation of the mandible.

Comparisons between T0 and T1 in group FM showed significant difference for SNA, ANB, Wits, Co-A, Co-Gn, NAP, A-Npog, overjet, and molar relation, indicating that at the end of treatment, the subjects in this group presented significantly higher values of these variables (Table 2). Whereas, in the comparisons between T0 and T1 in group MI, significant differences were shown for SNA, ANB, Wits, Co-A, Co-Gn, NAP, A-Npog, Sn-line H, 1-NB, overjet, and molar relation indicating that at the end of treatment, the subjects in this group presented significantly higher values of these variables (Table 3).

**Table 2:** Intragroup comparisons (T0 vs. T1) of the facial characteristics for the facemask group

Variable	T0 (baseline)		T1 (post-treatment)		*P* value*
	Median	Interquartile range	Median	Interquartile range	
SNA (°)	80.00	4.00	83.00	1.90	0.004
SNB (°)	82.00	2.00	82.77	2.47	0.673
ANB (°)	− 1.00	2.00	1.50	2.50	0.003
Wits (mm)	− 7.00	4.50	− 5.00	4.30	0.003
SN-GoGn (°)	32.50	5.80	33.50	7.50	0.166
Co-A (mm)	81.75	3,80	85.25	2.90	0.002
Co-Gn (mm)	112.00	7.50	116.00	18.00	0.002
NAP (°)	− 3.00	2.50	0.00	5.50	0.013
A-Npog (mm)	− 1.00	1.50	0.00	2.80	0.008
Sn-line H (mm)	5.00	2.30	4.75	2.30	0.726
1.NA (°)	29.00	7.30	30.00	4.50	0.875
1-NA (mm)	6.00	3.80	5.50	2.50	0.538
1.NB (°)	25.00	10.00	22.00	6.00	0.208
1-NB (mm)	5.00	4.30	4.50	4.00	0.267
Overjet (mm)	− 1.00	4.00	2.00	2.50	0.002
Overbite	1.00	2.80	2.00	1.80	0.123
MR (mm)	− 2.00	4.00	0.00	1.50	0.002

*Wilcoxon test. The results were expressed as median and interquartile range

**Table 3:** Intragroup comparisons (T0 vs. T1) of the facial characteristics for the mini-implant group

Variable	T0 (baseline)		T1 (post-treatment)		*P* value*
	Median	Interquartile range	Median	Interquartile range	
SNA (°)	84.00	5.40	86.75	4.60	0.002
SNB (°)	83.00	1.80	83.75	4.30	0.107
ANB (°)	− 1.00	4.00	1.50	2.50	0.005
Wits (mm)	− 6.50	3.80	− 2.75	4.50	0.002
SN-GoGn (°)	30.25	5.30	30.75	5.00	0.230
Co-A (mm)	85.75	10.90	89.50	12.80	0.002
Co-Gn (mm)	117.00	11.80	119.00	10.50	0.002
NAP (°)	− 2.00	8.40	1.50	7.00	0.013
A-Npog (mm)	− 0.50	3.40	1.00	3.40	0.008
Sn-line H (mm)	7.25	3.80	9.00	2.90	0.040
1.NA (°)	29.50	7.90	30.25	7.90	0.556
1-NA (mm)	6.50	5.40	6.50	2.60	0.559
1.NB (°)	28.25	5.80	27.00	7.30	0.413
1-NB (mm)	6.25	2.80	7.50	4.00	0.005
Overjet (mm)	− 3.00	4.00	2.00	3.00	0.003
Overbite (mm)	0.25	1.90	1.25	2.00	0.207
MR (mm)	− 2.00	4.80	1.25	4.30	0.002

*Wilcoxon test. The results were expressed as median and interquartile range

**Fig. 5:**
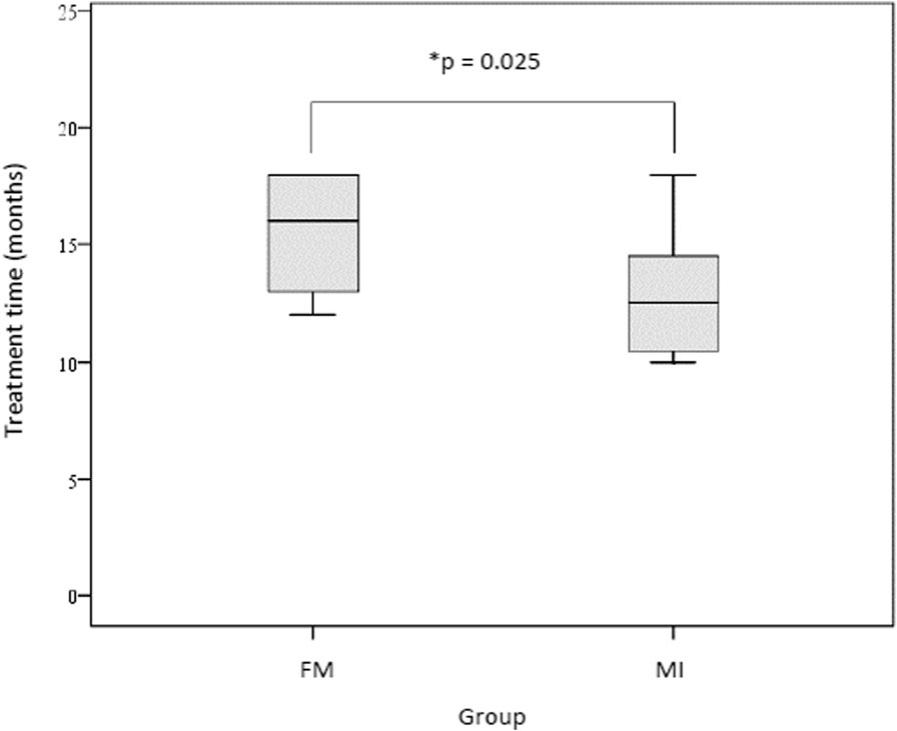
Time of treatment according to the groups. Height of the rectangle represents the first and third quartiles; the line that sections the rectangle represents the median; the semi-straight lines link quartiles 1 and 3 to the minimum and maximum values. **Mann-Whitney test

Comparative analysis of the changes in the facial characteristics showed no significant differences between groups FM and MI (Table 4). However, the median time of treatment in the group submitted to the technique with mini-implants was significantly shorter (12.5 vs. 16.0 months) (Fig. 5).

**Table 4:** Intergroup comparisons of the changes (T0–T1) in facial characteristics

Variable	Facemask		Mini-implant		*P* value*
	Median	Interquartile range	Median	Interquartile range	
SNA (°)	3.00	2.50	2.25	2.50	0.779
ANB (°)	2.00	2.50	2.00	3.30	0.978
Wits (mm)	3.00	4.00	2.75	4.80	0.398
Co-A (mm)	3.00	3.80	3.50	2.40	0.839
Co-Gn (mm)	3.00	3.50	2.50	5.50	0.956
NAP (°)	3.00	4.50	3.25	6.30	0.744
A-Npog (mm)	2.00	2.50	1.25	2.80	0.978
Sn-line H (mm)	0.25	3.40	1.50	2.30	0.073
1-NB (mm)	0.50	1.80	0.75	1.30	0.224
Overjet (mm)	2.00	3.00	3.00	3.50	0.679
MR (mm)	2.50	3.00	3.00	2.50	0.151

*Mann-Whitney test. The results were expressed as median and interquartile amplitude

### Stability of mini-implants

Of the 48 mini-implants installed in the 12 patients in the group MI, eight (16.7%) failed. The incidence of failure did not differ, according to the site of installation and sex of the patient; however, was higher in patients aged 11 to 12 compared to those aged 9 to 10 years (Table 5).

**Table 5:** Stability of the mini-implants, according to the sex, age, and installation site

Variable	Stable	Loss	*P value
	*n* (%)	*n* (%)	
Sex			
Male	15 (88.2%)	2 (11.8%)	0.694
Female	25 (80.6%)	6 (19.4%)	
Age			
9–10 years	23 (95.8%)	1 (4.2%)	0.048
11–12 years	17 (70.8%)	7 (29.2%)	
Site			
Maxilla	19 (79.2%)	5 (20.8%)	0.701
Mandible	21 (87.5%)	3 (12.5%)	

*Fisher’s exact test

## Discussion

After diverse authors [8, 10, 12, 14–18, 21] have demonstrated significant results with the use of implants and miniplates for maxillary protraction in class III patients, such as improvement in the facial profile, increase in convexity, and elimination of dental effects, some doubts arose during elaboration of the methodology of this study, in which the substitution of miniplates with mini-implants was proposed. Factors such as the stability of mini-implants with the use of higher forces and the biomechanics with intermaxillary elastics for protracting the maxilla raised the main questions. However, after a pilot study to evaluate the feasibility of the work, it was possible to visualize the improvement in facial and cephalometric measurements of the individuals treated with this protocol.

Some recent studies about anchorage with mini-implants for maxillary protraction corroborated the results obtained in the present research, and signaled that the biomechanics used and stability of the devices were sufficient to encourage studies with a larger sample to test the effectiveness of this therapeutic option [22–25]. However, some of these studies were presented in the form of case reports, other used removable orthodontic appliances, or facemask associated with mini-implants, and these evidently depended on greater cooperation from the patient. In one of these articles, RME was performed and the expander was used as anchorage in the maxillary arch, and mini-implants in the mandibular arch, evidently producing generally undesirable dental effects for the protocol with skeletal anchorage [24].

Therefore, the purpose of this clinical trial was to evaluate the use of conventional mini-implants in the maxilla and mandible as anchorage for maxillary protraction with intermaxillary elastics, in comparison with the protocol of RME/FM, which are the methods most used for treating class III patients with maxillary retrusion during facial growth. In addition to the good adhesion to this treatment option with intrabuccal elastics, other advantages of these temporary anchorage devices were the reduction in discomfort during the surgical procedure, low cost, and greater ease of insertion when compared with miniplates. These were the main justifications for testing mini-implants as one more orthopedic treatment option.

The stability of the mini-implants in some participants was the most critical point of the work, and it was essential for the effectiveness of the technique. As it was not possible to perform computed tomography in the children, the individual bone density was not analyzed, but it probably was the factor responsible for the loss of mini-implants in three of the 12 participants of group MI. Eight mini-implants were lost out of a total of 48 units. In these cases, three mini-implants were reinserted in one and the same individual (two in the maxilla and one in the mandible); three failures in two sites of insertion in the maxilla of another patient, and another two losses occurred in the maxilla and mandible of another child. There was no statistically significant difference between the sexes or the place of installation; however, children 9 and 10 years of age had more mini-implants stable during treatment than the larger ones (Table 5). This difference may be associated with supervision of oral hygiene of the parents, more carefully in the younger children. The devices were re-inserted 2 mm away from where they had been installed the first time, after a period of 2 months, to enable treatment to continue. The success rates of mini-implants and miniplates corresponded to approximately 80% [26, 27], a rate very similar to that which occurred in this study in which, only eight of the 48 mini-implants inserted needed to be reinserted (16.7%); however, as the procedure was not very invasive, it was well tolerated by the participants.

Encapsulation of the mini-implants in the oral mucosa tissue generally occurs when they are inserted outside of the attached gingiva region, and this is normally justified, in an attempt to reduce the risk of radicular perforation. This type of problem was not verified in any of the participants of this study. Miniplates present advantages in comparison with mini-implants, due to the possibility of inserting them further from the roots of permanent teeth by means of surgical access to the maxilla and mandible. Nevertheless, some authors have suggested that miniplates should not be inserted in patients before they are 10 years old, due to the presence of the mandibular canine germs [14]. In agreement with these authors, this was also the mean age of the sample of group MI, in which it was possible to insert the mini-implants. This ideal minimal age for treatment with temporary anchorage devices is in disagreement with some findings in patients who used the facemask. These authors emphasized that the most effective results occurred in patients under the age of 10 years [6].

Rapid maxillary expansion, a widely accepted treatment in the literature as an important stage in the treatment of class III, leads to destabilization of the maxillary sutures, optimizing maxillary protraction [28–30]. In recent studies related to correction of this malocclusion, in which groups with and without RME were analyzed, the authors demonstrated that this stage would not be mandatory before beginning with maxillary protraction with the facemask [31]. A study in which miniplates were used as anchorage for the facemask, the results proved that there were far fewer dental effects and vertical changes in the group without RME [32]. By virtue of this information, and as the participants of the present study presented no significant transverse changes, RME was not performed in the participants of group MI. Therefore, after placement of the mini-implants, it was possible to start immediate maxillary protrusion in the maxilla with intermaxillary elastics.

The cephalometric results obtained in the two groups reflected the improvement in facial profile and occlusion of the research participants. In Figs. 3a and 4, the effects of treatment in group MI were observed, particularly relative to the significant advancement of the maxilla: correction of the negative overjet, and balance between the upper and lower lip, reducing the concavity of the facial profile. In Tables 2 and 3, which show the intragroup comparison in T0 and T1, statistically significant differences occurred in the measurements SNA, ANB, Wits, Co-A, Co-Gn, NAP, A-Npog, overjet, and molar relation for both groups. In group MI, the measurements Sn-line H and 1-NB were also significant, which showed the improvement in facial profile and mandibular incisor protrusion, respectively. This change in the mandibular incisor may have been associated with the use of the stop made of flow resin to elevate the bite; although its protrusion was evident, it did not harm the maxillary correction by means of the technique with mini-implants used.

Table 4 and Fig. 3a, b show a similar pattern between the two techniques, indicating that there were no significant differences between groups FM and MI, in any of the cephalometric measurements in the two time intervals evaluated. In group FM, due to having a tooth-supported anchorage system by means of the expander, dental and skeletal effects, previously known results in the literature [2–7], evidently occurred, such as mesialization of the maxillary incisors and posterior movement of the mandibular incisors, increase in the mandibular plane and in the inferior height of the face, with rotation in the clockwise direction and increase in the facial pattern. In spite of not having been statistically significant in the two groups, it was possible to verify the increase in the SN.GoGn values in group FM, while in group MI, there was a mean reduction in these measurements (Tables 2 and 3), which showed different responses between the two techniques. As regards SNA, there were also no significant differences between the two groups; however, on an average, there was greater maxillary advancement in group MI than in group FM (Table 4).

All the results obtained in the present research using mini-implants, as well as other findings that showed the elastics adapted between maxillary and mandibular miniplates, had an inclination of approximately 45° in relation to the occlusal plane [33]. The elastics adapted to the hooks of the miniplates in the patients treated with facemask had an inclination of around 15° to 20° with the occlusal plane. Despite the differences in the anchoring and positioning devices of the elastics, the results showed that the two maxillary protraction procedures were effective and improved the facial concavity of class III patients. However, the groups treated with facemask presented a greater tendency of rotation of the mandibular plane in the clockwise direction, most probably due to differences in the point of application of force. While in the protocols with four skeletal anchoring devices, with elastic in a more vertical position contributed to the reduction of the mandibular rotation. In most of the cases analyzed in this study, there was a trend of anti-clockwise rotation, evidenced by the discrete differences in the palatine planes as shown in the patients’ overlaps. These differences are important for the correct indication of each protocol.

The force used in group MI, generated by the medium ¼′′ intermaxillary elastics, as from the second month, was 200 g on an average, and as in the studies with miniplates [10, 12, 14–18, 33], it was also localized below the center of resistance of the maxilla, but the anti-clockwise rotation of the palatine plane was discrete, without affecting the facial pattern of the patients. The different position of the mandibular mini-implants in group MI (Fig. 1), due to questions related to inter-radicular space for inserting the devices, apparently did not affect the response to treatment among the participants of this group.

Therefore, in this study it was possible to confirm the advantages that skeletal anchorage with mini-implants can offer in the treatment of patients with maxillary deficiency. This is another alternative for cases in which patients’ lack of cooperation with the use of the facemask occurs, or when there are psychosocial questions that affect the children. Moreover, there was a significantly shorter treatment time than that required for treatments with extraoral devices (Fig. 5). The ease of inserting mini-implants, lower cost, and greater comfort for the patient during placement surgery, in comparison with miniplates, must also be considered. These treatments may minimize the chances of having to undergo orthognathic surgery at the adult stage.

Even with so many advantages presented in this study, it is prudent to list the limitations, such as the impossibility of randomization of the sample, because it was necessary to evaluate the interradicular space for treatment of the MI group, increasing the risk of bias. The low prevalence of class III malocclusion made it difficult to standardize the sample according to the facial pattern. Therefore, further studies on this treatment protocol and evaluation of its long-term stability are required.

## Conclusion

- Conventional orthodontic mini-implants associated with intermaxillary elastics may be a treatment option for class III patients with maxillary retrusion.
- The most of the mini-implants remained stable during treatment.
- The mini-implant protocol reduced the undesirable effects of the conventional technique, within a shorter treatment time.

## Data Availability

All data generated or analyzed during this study are included in this article.

## Abbreviations

MI: Mini-implants
RME/FM: Rapid maxillary expansion and facemask
mm: Millimeter
′′: Inches
g: Gram
%: Percentage
1.NA: Angle between the long axis of the upper incisor and the NA line. Defines the inclination of the upper incisor relative to the maxilla
1.NB: Angle between the long axis of the lower incisor and the blade NB. Defines the inclination of the lower incisor relative to the mandible
1-NA: Linear measure between the most vestibular point of the upper incisor and the NA line. Defines the protrusion or retrusion of the upper incisor relative to the maxilla
1-NB: Linear measurement between the most vestibular point of the lower incisor and the line NB. Defines the protrusion or retrusion of the lower incisor relative to the mandible
ANB: Angle between the lines NA and NB. Represents the degree of sagittal discrepancy between the maxilla and mandible
Co-A: Distance between the points condyle and A. Represents the effective length of the middle face (maxilla)
Co-Gn: Distance between condyle and gnathic points. Defines the effective length of the mandible
Distance A-NP: Distance from point A to the line N-Pog. Describes the degree of convexity of the facial skeleton profile
MR: Molar relationship: distance between the mesial cusps of the first and second molars projected perpendicularly in the occlusal plane
NAP: Angle between the lines NA and APog. Describes the degree of convexity of the bone profile
OB: Overbite: distance between the incisal edges of the upper and lower central incisors measured perpendicular to the occlusal plane
OJ: Overjet: distance between the incisal edges of the upper and lower central incisors projected perpendicular to the occlusal plane
SN.GoGn: Angle between the SN and GoGn lines. Defines the direction of facial growth
SNA: Angle between the SN and NA lines. It represents the sagittal relation of the maxilla to the base of the skull
SNB: Angle between the lines SN and NB. Represents the sagittal relationship of the mandible to the base of the skull
Sn-Line H: Distance from the subnasal point to the line H. It describes the degree of convexity and harmony of the facial profile
T0: Baseline or initial time
T1: After time initial
vs: Versus
Witts: Distance between the perpendicular projections of points A and B on the functional occlusal plane. It defines the sagittal relationship between the maxilla and mandible
